# The causal association between circulating cytokines with the risk of frailty and sarcopenia under the perspective of geroscience

**DOI:** 10.3389/fendo.2024.1293146

**Published:** 2024-03-05

**Authors:** Congzhi Wang, Jiazhi Wang, Rui Wan, Hiroshi Kurihara, Min Wang

**Affiliations:** ^1^ Department of Internal Medicine Nursing, School of Nursing, Wannan Medical College, Wuhu, Anhui, China; ^2^ Sports Institute, Chi Zhou College, Chizhou, Anhui, China; ^3^ Business School, Yunnan University of Finance and Economics, Kunming, Yunnan, China; ^4^ Guangdong Engineering Research Center of Chinese Medicine and Disease Susceptibility/International Cooperative Laboratory of Traditional Chinese Medicine (TCM), Modernization, and Innovative Drug Development of Chinese Ministry of Education (MOE)/Guangdong Province Key Laboratory of Pharmacodynamic Constituents of Traditional Chinese Medicine (TCM) and New Drugs Research, Jinan University, Guangzhou, China; ^5^ Department of Pharmacy, Hainan General Hospital (Hainan Affiliated Hospital of Hainan Medical University), Haikou, Hainan, China

**Keywords:** causal association, circulating cytokines, frailty, geroscience, sarcopenia

## Abstract

**Introduction:**

Circulating cytokines were considered to play a critical role in the initiation and propagation of sarcopenia and frailty from observational studies. This study aimed to find the casual association between circulating cytokines and sarcopenia and frailty from a genetic perspective by two-sample Mendelian randomization (MR) analysis.

**Methods:**

Data for 41 circulating cytokines were extracted from the genome-wide association study dataset of 8,293 European participants. Inverse-variance weighted (IVW) method, MR-Egger, and weighted median method were applied to assess the relationship of circulating cytokines with the risk of aging-related syndromes and frailty. Furthermore, MR-Egger regression was used to indicate the directional pleiotropy, and Cochran’s Q test was used to verify the potential heterogeneity. The “leave-one-out” method was applied to visualize whether there was a causal relationship affected by only one anomalous single-nucleotide polymorphisms.

**Results:**

Genetic predisposition to increasing levels of interleukin-10 (IL-10), IL-12, and vascular endothelial growth factor (VEGF) was associated with the higher risk of low hand grip strength according to the IVW method [R = 1.05, 95% CI = 1.01–1.10, *P* = 0.028, false discovery rate (FDR)–adjusted *P* = 1.000; OR = 1.03, 95% CI = 1.00–1.07, *P* = 0.042, FDR-adjusted *P* = 0.784; OR = 1.02, 95% CI = 1.00–1.05, *P* = 0.038, FDR-adjusted *P* = 0.567]. Furthermore, genetically determined higher macrophage colony-stimulating factors (M-CSFs) were associated with a lower presence of appendicular lean mass (OR = 1.01, 95% CI = 1.00–1.02, *P* = 0.003, FDR-adjusted *P* = 0.103). Monokine induced by interferon-γ (MIG) and tumor necrosis factor–beta (TNF-β) were associated with a higher risk of frailty (OR = 1.03, 95% CI = 1.01–1.05, *P* < 0.0001, FDR-adjusted *P* = 0.012; OR = 1.01, 95% CI = 1.00–1.03, *P* = 0.013, FDR-adjusted *P* = 0.259). In this study, we did not find heterogeneity and horizontal pleiotropy between the circulating cytokines and the risk of frailty and sarcopenia.

**Conclusion:**

Genetic predisposition to assess IL-10, IL-12, and VEGF levels was associated with a higher risk of low hand grip strength and M-CSF with the presence of appendicular lean mass. The high levels of TNF-β and MIG were associated with a higher risk of frailty. More studies will be required to explore the molecular biological mechanisms underlying the action of inflammatory factors.

## Introduction

Currently, most countries around the globe face a demographic transition, 45% of the countries in the world are facing the problem of aging, with 12% of them facing severe aging (1). According to the latest data released by World Health Organization, as of February 2023, there will be approximately 1.3 billion older people in the world (2), and aging-related syndromes have emerged as the most important sub-health issue facing older people, damaging the social fabric and healthcare systems (2–5). Aging is an inevitable proposition for every individual and is a multifaceted process that includes a wide range of phenotypes such as sarcopenia, frailty, and epigenetic senescence (6, 7). As a novel conception enrolled in aging in recent years, the systemic inflammatory state of the aging immune system is termed ‘‘inflammaging” (8), which indicates that inflammation was pathogenesis in the progression of aging. “Inflammaging” is described as a state of low-grade, long-term, aseptic inflammation that occurs with aging (9, 10). It is very prevalent in the geriatric population and is characterized by increased levels of blood markers of inflammation, leading to an elevated rate of exposure to chronic disease, frailty, disablement, and preterm death (11–13). However, early detection of specific cellular and molecular biomarkers of aging can help identify aging-related syndromes, and advanced interventions can help reduce the incidence of aging syndromes in at-risk populations (9).

Sarcopenia, or “muscle loss,” was described sarcopenia as “a progressive, aging-related, generalized loss of muscle mass and muscle strength or a reduction in muscle function” (14), which is one of the essential clinical manifestations of aging-related syndromes (15). During the past decades, the notion of sarcopenia has evolved into a more comprehensive system comprising the relevant indicator parameters and functions; the criteria of sarcopenia are the loss of skeletal muscle mass, less muscle strength, and lower physical ability than healthy elderly (16, 17). A total of 0.8%–0.98% of men had sarcopenia, whereas 0.64%–0.7% of women suffered from the same muscle loss (17, 18). Sarcopenia not only reduces the quality of life of the elderly but also interferes with daily activities (19), increases susceptibility to various diseases and the burden of healthcare on society, and leads to a strain on healthcare resources (20). These epidemiological investigations have shown that circulating cytokines were significantly associated with sarcopenia (21, 22). These studies showed that interleukin-6 (IL-6) and tumor necrosis factor–alpha (TNF-α) were related to the risk of sarcopenia (23, 24), whereas other studies showed the risk factor of circulating cytokines was TNF-α, IL-6, IL-8, IL-15, and monocyte chemotactic protein-1 (MCP-1) was demonstrated as a risk factor for sarcopenia (25–27). The association between sarcopenia and circulating cytokines remains controversial; thus, we still need to perform Mendelian randomization (MR) analysis to identify the association.

As another essential feature of aging, frailty was defined as a widespread decrease in physiological functioning in older adults (28), a status of enhanced susceptibility to stressful pressures, a multi-system imbalance unrelated to aging in time or diseases (29), and a predisposition to many adverse health-related incidents (30). Frailty was strongly associated with some chronic diseases of the elderly, which encompass cognitive impairment, Parkinson’s disease, and chronic cardiovascular disease (heart failure and atrial fibrillation) (31), which leads to a lower quality of life for older people and a lower sense of well-being in life, resulting in higher rates of mortality and disability (32–34). A systematic review revealed that IL-6, IL-1β, and TNF-α were inflammatory predictors of frailty (35). Whereas, other studies suggested that C-reaction protein, IL-6, and TNF-α were positively associated with the risk of frailty (36–38). In addition, growth and differentiation factor 1, IL-8, IL-1β, interferon-γ (IFN-γ), chemokine regulated upon activation, normal T-cell expressed and secreted (RANTES), and MCP-1 were demonstrated to play an essential role in intercellular communication and promote the progression of aging from other studies (39, 40). However, because of the large bias between these results of traditional observational studies, it is difficult for us to ascertain which specific risk factors have potential for the frailty. In this context, identifying putative biomarkers was essential for slowing the progress of aging, and recognizing the hallmark of aging-related syndromes and aging as foundations for healthy aging.

Some conventional epidemiological studies revealed the correlation between circulating cytokines and aging-related traits; however, traditional studies cannot remove bias due to confounding variables measurement error, and bidirectional causality (41). Moreover, aging-related traits have diverse clinical presentations and accumulate in multiple systems and organs, making it challenging to identify the relationship between circulating cytokines and aging-related traits through traditional methods. Thus, the MR study provides an randomized controlled trial (RCT) research method (42); meanwhile, the MR analysis avoids the very high difficulty of RCTs, which require a lot of money and manpower, as well as moral and ethical review (43, 44). Hence, we will systematically study from a genetic perspective, which can avoid the interference of confounding factors and reverse causality from conventional epidemiological studies (45, 46).

## Methods

### Study overview

**Figure 1:**
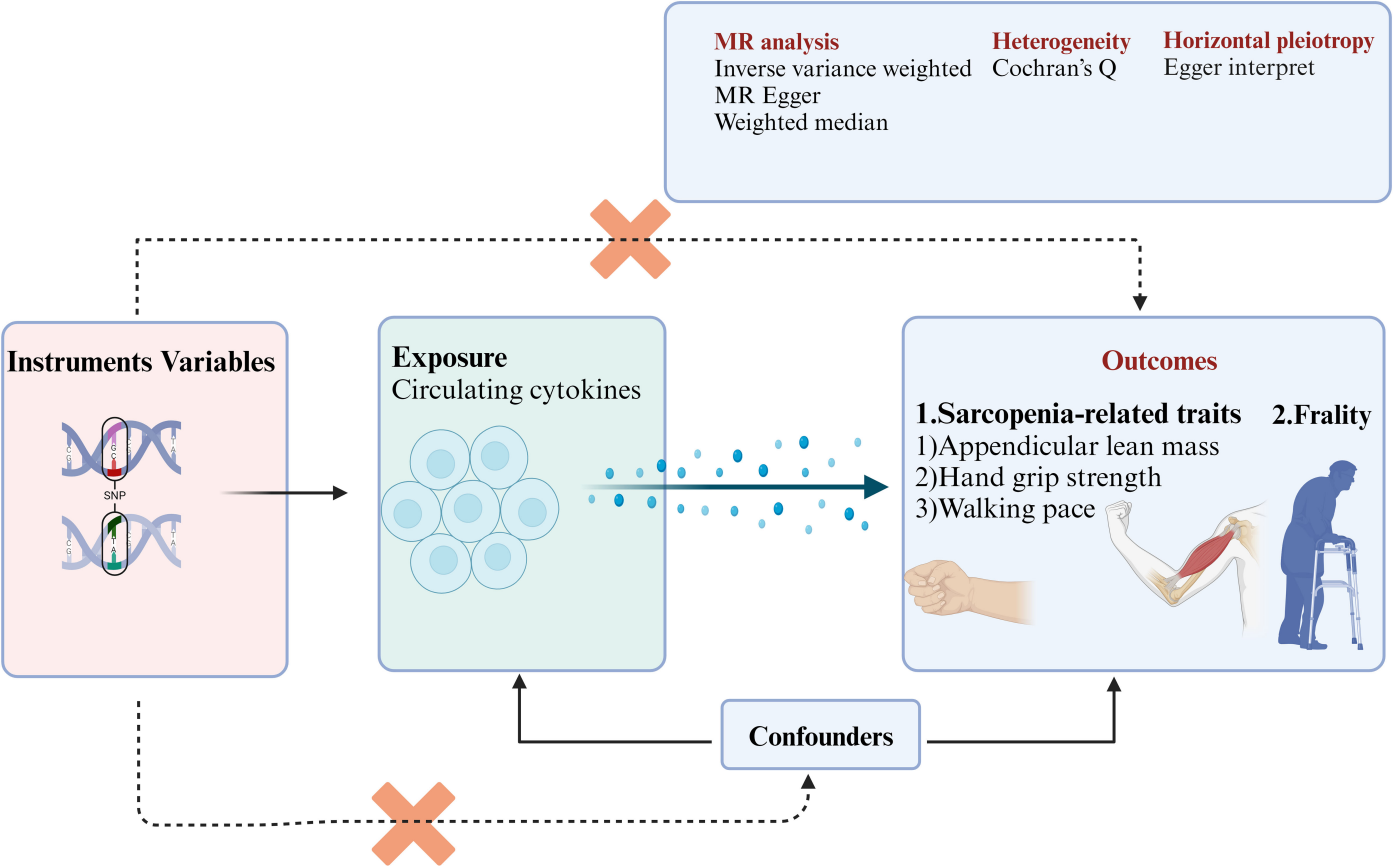
The flowchart of this study enrolled in the MR study. SNPs, single-nucleotide polymorphisms; MR, Mendelian randomization.

The purpose of the study was to indicate a systematic and comprehensive analysis of the circulating cytokines that are causally associated with aging-related syndromes. The design flowchart and research assumption are presented in **Figure 1**. (1) In the MR study, we find specific single-nucleotide polymorphisms (SNPs) as instrument variables and strongly associated with the 41 circulating cytokines. (2) The 41 circulating cytokines were significant with the outcomes including aging-related syndromes (three sarcopenia-related traits and frailty). (3) The SNPs were not correlated with outcome variables and can only influence outcome variables through exposure variables. The instrument variables were not affected by the confounder’s factors. The open genome-wide association study (GWAS) summary statistics were used from the GWAS websites (https://gwas.mrcieu.ac.uk/), and ethical approval was not needed in the study (41, 47, 48). This study is reported following the Strengthening the Reporting of Observational Studies in Epidemiology Using Mendelian Randomization guidelines (48).

### Genetic instruments for circulating cytokines

The data of circulating cytokines were extracted from the datasets of GWAS, which enrolled 8,293 European participants (49). Detailed information on circulating cytokines is presented in **Additional File**, **Supplementary Table S1**. The Cardiovascular Risk in Young Finns Study is a multicenter follow-up study with randomly chosen subjects from the Finnish cities of Helsinki, Kuopio, Oulu, Tampere, and Turku and their rural surroundings. The study began in 1980 when 3,596 children and young adults participated in the first cross-sectional survey. The follow-up visits have been conducted in 1983, 1986, 1989, 2001, 2007, and 2011. The present cross-sectional study includes 2,019 unrelated individuals who participated in the 2007 follow-up and who had both cytokines’ measurements and genotype data available. In addition, gene expression data from 1,664 participants of the 2011 follow-up were analyzed for the present study. All participants gave a written informed consent, and the study was approved by local ethics committees. The conditions for selecting an SNP as a strong instrument are all SNPs that strongly and independently (r^2 = ^0.001, distance = 10,000 kb) predicted cytokines at genome-wide significance (*P* < 5×10^−6^). After, 145 SNPs were selected to use for the MR analysis. The study analyzed cytokines data from investigators in 1997 and 2002 in a random sample of subjects aged 25–74 years from five regions of Finland every 5 years, to monitor the level of chronic disease risk factors in Finland. Peripheral blood samples were collected after the investigators signed an informed consent form. Forty-eight cytokines were examined in the peripheral blood samples using a Bio-Rad’s premixed Bio-Plex Pro assay kit. Only values within the cytokines-specific detection range were included in the analysis, and cytokines with more than 90% missing values were excluded.

### Genetic instruments for sarcopenia-related traits

According to the Asian Working Group for Sarcopenia (AWGS) in 2019, the sarcopenia was diagnose with three critical traits: Traits 1: low handgrip strength, men < 28 kg, women < 18 kg; traits 2: low muscle mass, men < 7.0 kg/m^2^, women < 5.4 kg/m^2^; traits 3: usual walking pace, gait speed < 1.0 m/s, chair stand test ≥ 12 s. Three sarcopenia-related traits were used as outcome variables including low hand grip strength, appendicular lean mass, and usual walking pace. The GWAS summary data of low hand grip strength was acquired from the meta-analysis study including 48,596 low hand grip strength cases and 207,927 controls of the European population (PMID:33510174). Low hand grip strength was by hand grip strength (EWGSOP definition: men’s grip strength was less than 30 kg; women’s grip strength was less than 20 kg) (50). Genome-wide significant (*P*< 5×10^−6^) SNPs were retained for low hand grip strength. After clumping (r^2 = ^0.001, distance = 10,000 kb), and 420 SNPs were left as independent Instruments Variables (IVs) for low hand grip strength. The GWAS summary data of appendicular lean mass were obtained from the study including 450,243 cases. The age of participants ranged from 38 to 70, which was composed of 244,730 female and 205,513 male participants (PMID: 33097823) (51). Genome-wide significant (*P* < 5 × 10^−6^) SNPs were retained for low hand grip strength. After clumping (r^2 = ^0.001, distance = 10,000 kb), 431 SNPs were left as independent IVs for appendicular lean mass. The GWAS summary data of the usual walking pace were obtained from the study including 459,915 samples (ukb-b-4711). Ten genome-wide significant (*P* < 5 × 10^−6^) SNPs were retained for low hand grip strength. After clumping (r^2 = ^0.001, distance = 10,000 kb), 411 SNPs were left as independent IVs for low hand grip strength.

### Genetic instruments for frailty

The GWAS summary data of frailty were acquired from the meta-analysis study including 164,610 United Kingdom (UK) Biobank participants, 10,616 TwinGene participants, and 368 SATSA Swedish Adoption/Twin Study of Aging population participants (PMID: 34431594) frailty (52). Genome-wide significant (*P* < 5 × 10^−6^) SNPs were retained for frailty. After clumping (r^2 = ^0.001, distance = 10,000 kb), 388 SNPs were left as independent IVs for frailty. The frailty index was calculated as the ratio of the number of deficiencies present to the total number of possible deficiencies, resulting in a continuous score ranging from perfectly healthy (0) to perfectly frail (1). Thus, a total of 46 frailty indices were constructed (including deficiencies associated with disability, comorbidities, symptoms, and irregular laboratory values). Frailty indices are intended to be used as continuous scores but can also be categorized on the basis of recommended critical scores determined using specific stratified likelihood ratios. A frailty index of 0.10 is “non-frailty,” 0.10 < FI ≤ 0.21 is “pre-frailty,” 0.21 < FI ≤ 0.45 is “frailty,” and FI > 0.45 is “most frailty”.

### MR analysis

The screening SNPs information of 41 circulating cytokines was extracted from the datasets and was listed in **Additional File**, **Supplementary Table S1**, including the effect allele, other alleles, frequency, standard error *P*-value, and the total number of SNPs. The index of *F* was used to evaluate the strength of instruments (*F >*10) (53). Inverse-variance weighted (IVW) method was the main method to assess the relationship of circulating cytokines with the risk of aging-related syndromes (54). Furthermore, MR-Egger and weighted median were performed for MR analysis. In addition, MR-Egger regression was used to indicate the directional pleiotropy, if *P*-values = 0.05 were regarded as the threshold value for directional pleiotropy; Cochran’s Q test was used to verify the potential heterogeneity (47). Furthermore, the “leave-one-out” method was applied to visualize whether there was a causal relationship affected by only one anomalous SNP (44). The scatter plots represent the causal association between the circulating cytokines on the risk of sarcopenia-related traits. Packages “Devtools” is used for making developing R packages easier, Packages “MRPRESSO” is used to perform the MR pleiotropy residual sum and outlier test, Packages “Two Sample MR” is used for two samples MR functions and interface to MR base database, and “MR Instruments” were run for data sources for genetic instruments to be used in MR. All the analysis was conducted in R studio. To decrease the chances of occurrence of false positives, the false discovery rate (FDR) adjusted *P*-values is calculated to correct for multiple comparisons.

## Results

### Casual effect of circulating cytokines on the risk of sarcopenia-related traits

**Figure 2:**
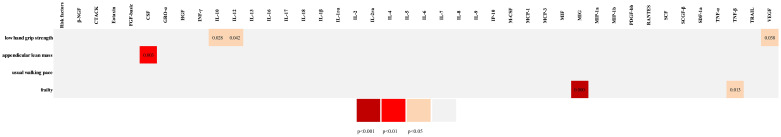
The overall Mendelian randomization association between circulating cytokines and frailty and sarcopenia. β-NGF, beta nerve growth factor; CTACK, cutaneous T-cell attracting (CCL27); FGF-basic, basic fibroblast growth factor; CSF, colony-stimulating factor; GRO-a, growth-regulated oncogene-α (CXCL1); HGF, hepatocyte growth factor; IFN-γ, interferon-gamma; IL-10, interleukin-10; IL-12, interleukin-12; IL-13, interleukin-13; IL-16, interleukin-16; IL-17, interleukin-17; IL-18, interleukin-18; IL-1rα, interleukin-1 receptor antagonist; IL-1β, interleukin-1-beta; IL-2, interleukin-2; IL-2rα, interleukin-2 receptor, alpha subunit; IL-4, interleukin-4; IL-5, interleukin-5; IL-6, interleukin-6; IL-7, interleukin-7; IL-8, interleukin-8; IL-9, interleukin-9; IP-10, interferon gamma-induced protein 10 (CXCL10); CSF, colony-stimulating factor; MCP-1, monocyte chemotactic protein-1 (CCL2); MCP-3, monocyte-specific chemokine 3 (CCL7); MIF, macrophage migration inhibitory factor; MIG, monokine induced by interferon-gamma; MIP-1α, macrophage inflammatory protein-1α (CCL3); MIP-1b, macrophage inflammatory protein-1β; PDGF-bb, platelet-derived growth factor BB; RANTES, regulated on activation, normal T-cell expressed and secreted (CCL5); SCF, stem cell factor; SCGF-β, stem cell growth factor beta; SDF-1α, stromal cell–derived factor-1 alpha; TNF-α, tumor necrosis factor–alpha; TNF-β, tumor necrosis factor–beta; TRAIL, TNF-related apoptosis-inducing ligand; VEGF, vascular endothelial growth factor. SNP, single-nucleotide polymorphism; OR, odds ratio.

**Figure 3:**
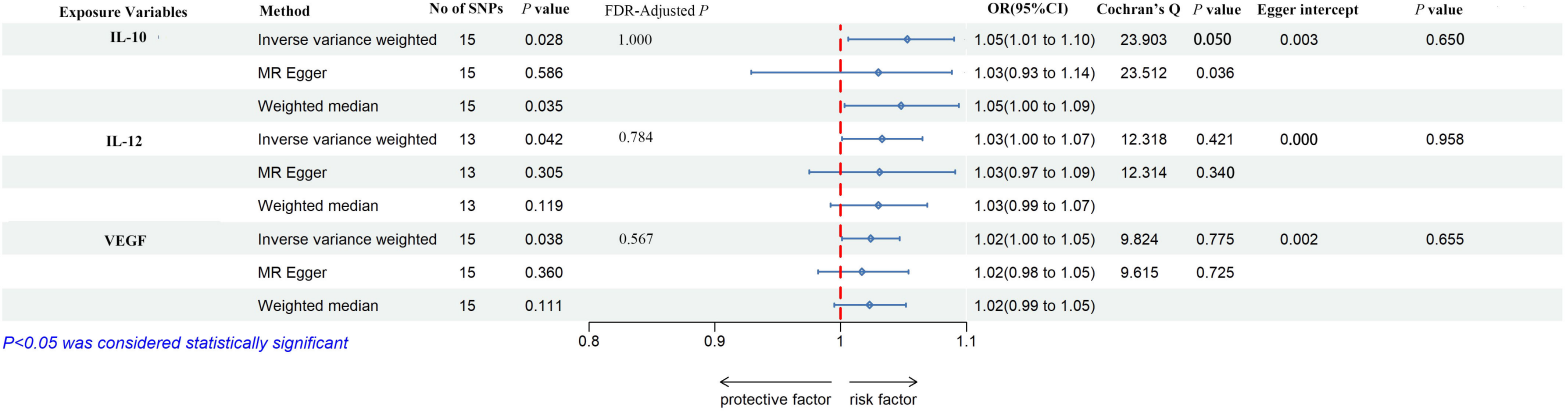
The forest plot of the association between circulating cytokines and low hand grip strength performed by MR analysis. Three methods were used for MR analysis, which were inverse variance weighted, MR-Egger, and weighted median, with OR value representing the association between circulating cytokines and low hand grip strength. FDR adjustment was used to adjust the *P-*value. In addition, Cochran’s Q was used to assess heterogeneity, and Egger interpret was used to test horizontal pleiotropy. IL-10, interleukin-10; IL-12, interleukin-12; VEGF, vascular endothelial growth factor; SNPs, single-nucleotide polymorphisms; OR, odds ratio; FDR, false-discovery rate; CI, confidence interval.

**Figure 4:**
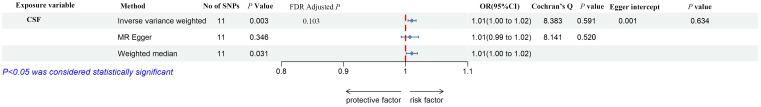
The forest plot of the association between circulating cytokines and the presence of appendicular lean mass performed by MR analysis. Three methods were used for MR analysis, which were inverse variance weighted, MR-Egger, and weighted median, with OR value representing the association between circulating cytokines and low hand grip strength. FDR adjustment was used to adjust the *P*-value. In addition, Cochran’s Q was used to assess heterogeneity, and Egger interpret was used to test horizontal pleiotropy. CSF, colony-stimulating factor; SNPs, single-nucleotide polymorphisms; OR, odds ratio; FDR, false-discovery rate; CI, confidence interval.

As shown in **Figures 2**, **3**, IL-10, IL-12, and VEGF were significant with a higher risk of low hand grip strength based on the IVW method. IL-10 was identified to be causally associated with low hand grip strength (OR = 1.05, 95% CI = 1.01–1.10, *P* = 0.028, FDR-adjusted *P* = 1.000), as well as IL-12 (OR = 1.03, 95% CI = 1.00–1.07, *P* = 0.042, FDR-adjusted *P* = 0.784), and VEGF (OR = 1.02, 95% CI = 1.00–1.05, *P* = 0.038, FDR-adjusted *P* = 0.567). As shown in **Figures 2**, **4**, macrophage colony-stimulating factor (M-CSF) was associated with appendicular lean mass (OR = 1.01, 95% CI = 1.00–1.02, *P* = 0.003, FDR-adjusted *P* = 0.103). However, no significant association was found between circulating cytokines and the usual walking pace. All results are shown in **Additional File**, **Supplementary Tables S2**-**S4**.

According to Cochran’s Q test and MR-Egger test, the heterogeneity and horizontal pleiotropy of this study were tested respectively. As shown in **Figure 3**, there was no heterogeneity between IL-10 and low hand grip strength (Cochran’s Q-statistic = 23.903, *P* = 0.050), as well as horizontal pleiotropy (Egger intercept = 0.003, *P* = 0.650). IL-12 also has no heterogeneity and horizontal pleiotropy (Cochran’s Q-statistic = 12.318, *P* = 0.421) (Egger intercept = 0.000, *P* = 0.958), as well as VEGF (Cochran’s Q-statistic = 9.824, *P* = 0.775) (Egger intercept = 0.002, *P* = 0.655). As shown in **Figure 4**, there was no heterogeneity between M-CSF and the appendicular lean mass (Cochran’s Q-statistic = 8.383, *P* = 0.591), as well as horizontal pleiotropy (Egger intercept = 0.001, *P* = 0.634). Scatter plots of SNPs effect size estimate were displayed in **Figures 6A**–**D**, which showed the association between circulating cytokines and risk of sarcopenia predicted by three different MR methods. The scatter plots were displayed in **Figures 7A**–**D**, which showed the relationship between inflammatory cytokines and the risk of sarcopenia-related syndromes. In the results of the leave-one-out test, there were no SNPs with large effect sizes that were biased in their estimates from **Figures 8A–D**.

### Casual effect of circulating cytokines on the risk of frailty

**Figure 5:**
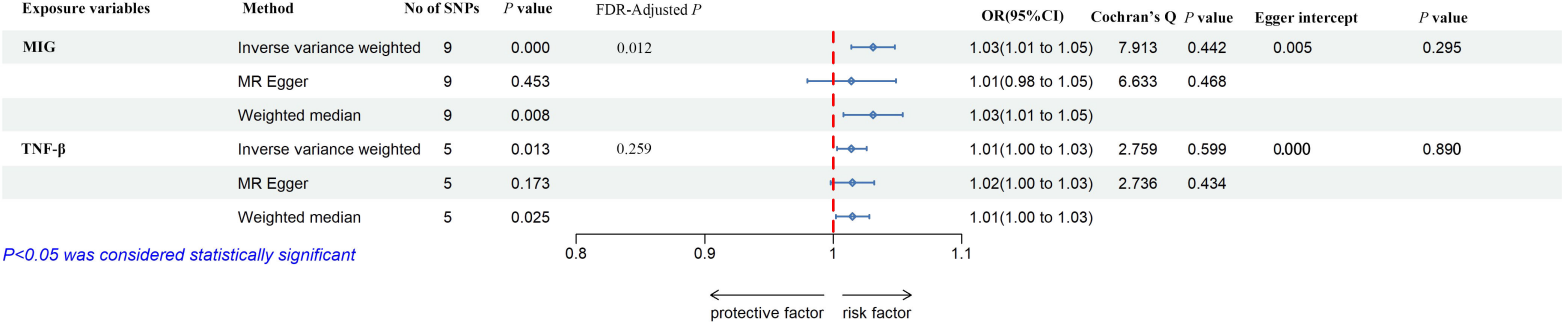
The forest plot of the association between circulating cytokines and frailty by MR analysis. Three methods were used for MR analysis, which were inverse variance weighted, MR-Egger, and weighted median, with OR value representing the association between circulating cytokines and low hand grip strength. FDR adjustment was used to adjust the *P*-value. In addition, Cochran’s Q was used to assess heterogeneity, and Egger interpret was used to test horizontal pleiotropy. MIG, monokine induced by interferon-gamma; tumor necrosis factor–beta. TNF-β, tumor necrosis factor–beta; SNPs, single-nucleotide polymorphisms; OR, odds ratio; FDR, false-discovery rate; CI, confidence interval.

**Figure 6:**
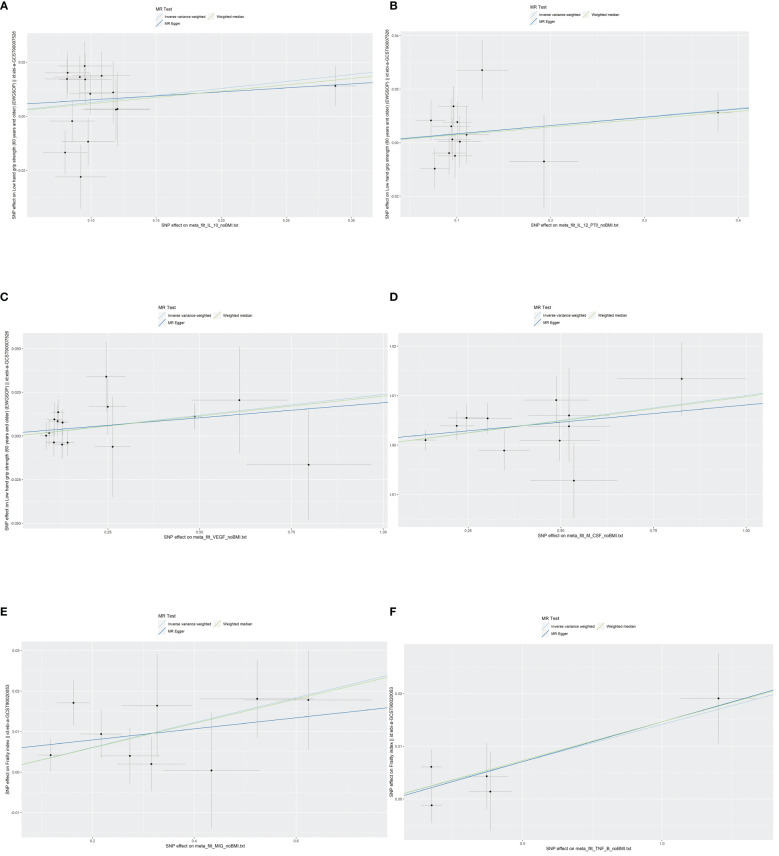
Scatter plots of SNPs effect size estimate. Genetic associations between circulating cytokines and risk of frailty and sarcopenia predicted by three different MR methods. Horizontal coordinates showed associations of SNPs with circulating cytokines, vertical coordinates showed associations of SNPs with frailty and sarcopenia, each dot represented SNPs, and two short lines on the dots showed the 95% CIs for the effect of circulating cytokines on the values for frailty and sarcopenia. **(A)** Association between IL-10 and the risk of low hand grip strength. **(B)** Association between IL-12 and the risk of low hand grip strength. **(C)** Association between VEGF and the risk of low hand grip strength. **(D)** Association between M-CSF and the risk of the presence of appendicular lean mass. **(E)** Association between MIG and the risk of frailty. **(F)** Association between TNF-β and the risk of frailty. MR, Mendelian randomization; IL-10, interleukin-10; IL-12, interleukin-12; VEGF, vascular endothelial growth factor; CSF, colony-stimulating factor; MIG, monokine induced by interferon-gamma; TNF-β, tumor necrosis factor–beta; SNPs, single-nucleotide polymorphisms.

**Figure 7:**
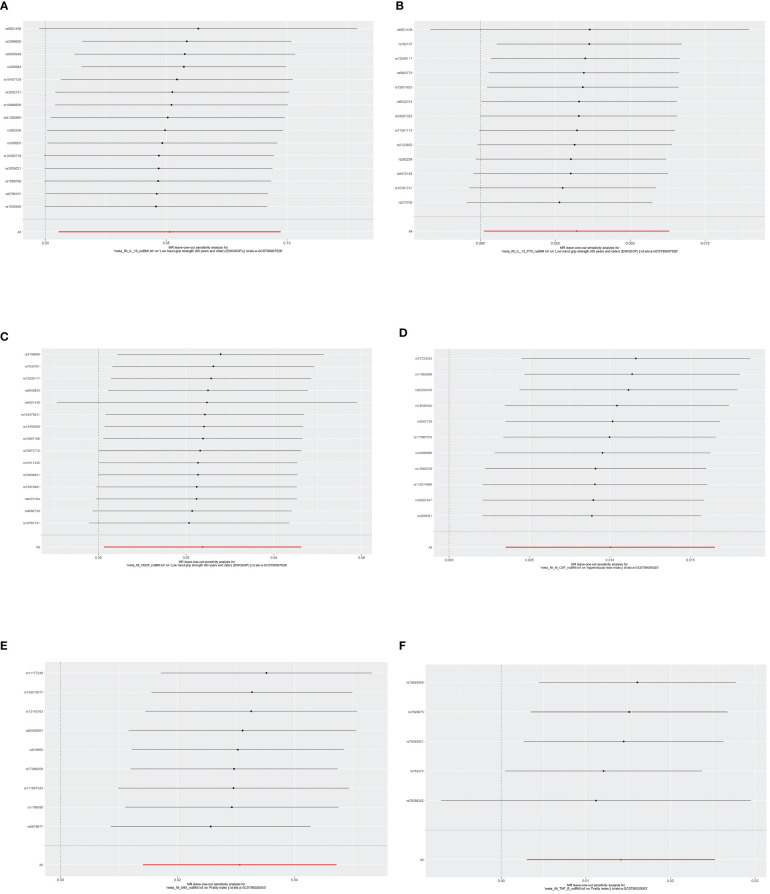
The results of leave-one-out analyses. The horizontal axis showed leave-one-out sensitivity analysis for circulating cytokines and risk of age-related syndromes. The vertical axis indicated the effect of each SNP on frailty and sarcopenia. **(A)** The leave-one-out sensitivity analysis for IL-10 and the risk of low hand grip strength. **(B)** The leave-one-out sensitivity analysis for IL-12 and the risk of low hand grip strength. **(C)** The leave-one-out sensitivity analysis for VEGF and the risk of low hand grip strength. **(D)** The leave-one-out sensitivity analysis for M-CSF and the risk of the presence of appendicular lean mass. **(E)** The leave-one-out sensitivity analysis for MIG and the risk of frailty. **(F)** The leave-one-out sensitivity analysis for TNF-β and the risk of frailty. MR, Mendelian randomization; IL-10, interleukin-10; IL-12, interleukin-12; VEGF, vascular endothelial growth factor; CSF, colony-stimulating factor; MIG, monokine induced by interferon-gamma; TNF-β, tumor necrosis factor–beta; SNPs, single-nucleotide polymorphisms.

**Figure 8:**
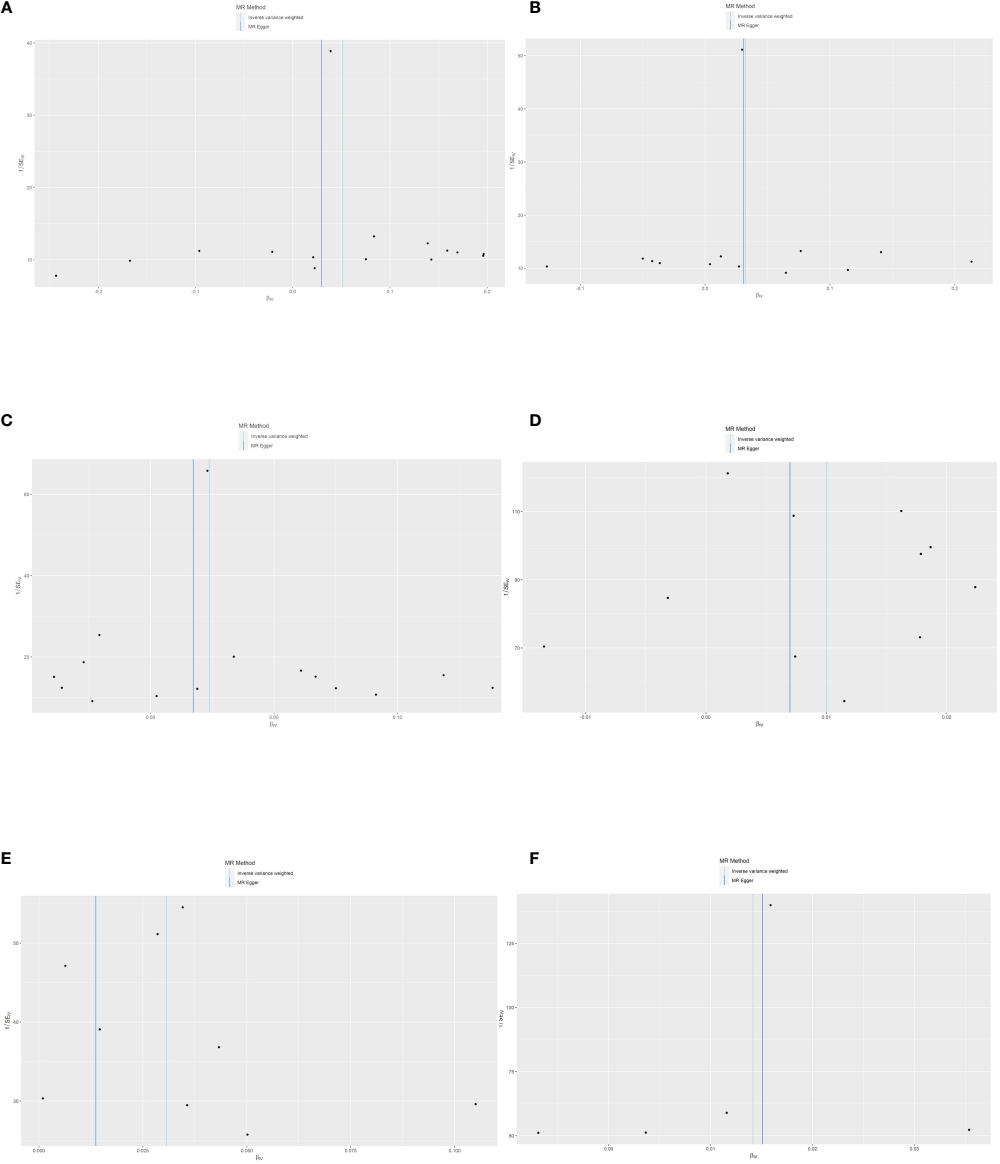
The funnel plot of SNPs. Each point was the SNP, MR-Egger, and inverse variance weighted method that were used to assess the heterogeneity. **(A)** The heterogeneity evaluation of IL-10 and the risk of low hand grip strength. **(B)** The heterogeneity evaluation of IL-12 and the risk of low hand grip strength. **(C)** The heterogeneity evaluation of VEGF and the risk of low hand grip strength. **(D)** The heterogeneity evaluation of M-CSF and the risk of appendicular lean mass. **(E)** The heterogeneity evaluation of MIG and the risk of frailty. **(F)** The heterogeneity evaluation of TNF-β and the risk of frailty. MR, Mendelian randomization; IL-10, interleukin-10; IL-12, interleukin-12; VEGF, vascular endothelial growth factor; CSF, colony-stimulating factor; MIG, monokine induced by interferon-gamma; TNF-β, tumor necrosis factor–beta; SNP, single-nucleotide polymorphisms.

As shown in **Figures  2**, **5**, MIG was significant with a higher risk of frailty based on the IVW method (OR = 1.03, 95% CI = 1.01–1.05, *P* < 0.0001, FDR-adjusted *P* = 0.012), as well as TNF-β (OR = 1.01, 95% CI = 1.00–1.03, *P* = 0.013, FDR-adjusted *P* = 0.259). All results are shown in **Additional File**, **Supplementary Table S5**. As shown in **Figure 4**, no heterogeneity or horizontal pleiotropy was observed in the study (Cochran’s Q-statistic = 7.913, *P* = 0.442) (Egger intercept = 0.005, *P* = 0.295). Scatter plots of SNPs effect size estimate were displayed in **Figures 6E**, **F**, which showed the association between circulating cytokines and risk of frailty predicted by three different MR methods. The same results were present in TNF-β (Cochran’s Q-statistic = 2.759, *P* = 0.599) (Egger intercept = 0.000, *P* = 0.890). The scatter plots were displayed in **Figures 7E, F**, which showed the relationship between inflammatory cytokines and the risk of frailty. In the results of the leave-one-out test, there were no SNPs with large effect sizes that were biased in their estimates from **Figures 8E, F**.

## Discussion

Two-sample MR study was used to explore the associations with circulating cytokines, frailty, and sarcopenia. To the best of our knowledge, this is the first study to indicate casual relationships for the first time from a genetic perspective systematically. We adopted three traits for the sarcopenia including usual walking pace, low hand grip strength, and appendicular lean mass in the study. Regarding the 41 cytokines, we found that the IL-10, IL-12, and VEGF were potentially associated with a higher risk of low hand grip strength, and CSF was also potentially correlated with a higher risk of appendicular lean mass. However, no circulating cytokines were found associated with a higher risk of the usual walking pace. In addition, we further predicted MIG was significantly associated with correlated with frailty, whereas TNF-β was potentially correlated with a higher risk of frailty.

Genetic predisposition IL-10, IL-12, and vascular endothelial growth factor (VEGF) levels were potentially associated with a higher risk of low hand grip strength in **Figure 3**. IL-10 is a risk factor for low hand grip strength, which was inconsistent with these observational studies (55–59). The mechanism was obscure, needed more large-sample studies, and excluded confounding factors. A positive correlation was reported between IL-12 and low hand grip strength. IL-12 is an interleukin that is naturally secreted by macrophages, neutrophils, dendritic cells, and human B cells in response to antigen stimulation (60). IL-12 is a member of the IL-12 gene group, which is the only family of heterodimeric cytokines (61). A study conducted by Chen et al. found that the decreasing level of IL-12 was strongly related to the occurrence of sarcopenia (OR = 0.36, 95% CI = 0.150–0.834) (60). However, Jung et al. indicated that lower inhibition of IL-12/23 was a protective role for muscle atrophy in the colitis-induced trial. The association between the circulating cytokines and sarcopenia remains controversial, and the demographic differences contribute to this difference in conclusions (62). Thus, more large-sample studies need to be conducted to further validate the relationship. VEGF was proven to be a protective factor in mice, can prevent age-related losses of capillaries, enhances the perfusion and function of organs, and extends life span (63). However, there were no studies on the potential role of VEGF on sarcopenia in humans. In the MR study, the evidence supported that VEGF was a risk factor for low hand grip strength for the first time.

This study genetically predicted a potential association between circulating CSFs (colony-stimulating factors) and the lower presence of appendicular lean mass in **Figure 4**. CSFs are secreted glycoproteins that bind to receptor proteins on the surfaces of hematopoietic stem cells, thereby activating intracellular signaling pathways that can cause the cells to proliferate and differentiate into a specific kind of blood cell, usually white blood cells (64, 65). Ohashi et al. demonstrated that CSF is involved in load-induced muscle hypertrophy in mice and suggested that CSF is a potential agent for the treatment of patients with sarcopenia (66). Kowalski et al. found that the combination of Sdf-1 and CSF significantly improved the skeletal muscle regeneration and proliferation of skeletal muscle. Sdf-1 and CSF treatment increased the number of monocytes associated with myofibers, by increasing the counts of CD34+ and Cxcr4+ cells as well as the expression of Cxcr7 (67). In this context, more studies on whether CGF increases weight and related functions in skeletal muscle require further validation in humans.

As shown in **Figure 5**, the study genetically predicted that TNF-β was recognized as risk factor for frailty, and the study supports the potential association. TNF-β is an important mediator and belongs to the TNF superfamily of ligands, which are strongly associated with inflammation (68). Whereas, some epidemiological studies indicated that proinflammatory cytokines are associated with frailty was TNF-α (69–75), which was inconsistent with our study. These studies supported that TNF-α and IL-6 are proinflammatory cytokines, can alter intercellular communication, and affect the differentiation of muscle cells and induction of apoptosis (70–74). In addition, these inflammatory frailty biomarkers can indirectly contribute to physical weakness through the endocrine and musculoskeletal systems, leading to nutritional imbalances and damage to the cardiovascular system (75). TNF-α and TNF-β are different members of the TNF superfamily. TNF produced by macrophages is named TNF-α, naming the lymphotoxin [lymphotoxin (LT)] produced by T lymphocytes as TNF-β (76). The biological roles of TNF-α and TNF-β are extremely similar, which may be related to similarities in molecular structure and receptor identity (77). Nevertheless, the study found merely a causal relationship between TNF-β and frailty, not TNF-α, which was not reported in the previous studies. We hypothesize that this difference comes mainly from confounding factors such as demography, we found that these study samples were middle-aged and elderly people, and frail people tend to suffer from various chronic diseases, which also include chronic inflammatory diseases that do not have any clinical manifestations (78). Therefore, it is difficult to exclude the interference of these factors in traditional epidemiological studies, and the present study is the first MR study that evaluates the causal relationship between inflammatory factors and debilitation from a genetic viewpoint, which may be the reason why this study is different from other studies.

The study genetically predicted that MIG was significantly associated with correlated with frailty; MIG is an abbreviation for the abbreviation of monokine induced by IFN-γ, which was called chemokine (C-X-C motif) ligand 9 (CXCL9). MIG is induced early in the response to IFN-γ. MIG is a T-cell chemokine, and IFN-γ induces MIG expression. Liang et al. found that our results indicate a potential effect of serum MIG on the progression of atherosclerosis and suggest that MIG may be a useful biomarker of the severity of coronary artery disease (79). The mechanism is ambiguous and needs to be justified by further research. Currently, there are different criteria for diagnosis, which is one of the main reasons for the differences in the results. MIG was identified as a risk factor among the 41 circulating cytokines, becoming a new direction in the study of frailty from inflammation, and it has never been found in previous studies.

This study includes the following advantages. First, no studies have systematically evaluated the relationship between inflammatory factors and sarcopenia and frailty from a genetic perspective, 41 circulating cytokines were included in this study as exposure variables, and this study elaborated on the relationship from the perspective of inflammatory factors and provided an important reference for future treatment and nursing. Second, there is no pleiotropy and heterogeneity in the dataset of this study, which indicates that the results have good reliability and stability. Third, these results will help inform future studies designed to promote healthy aging and provide new mechanisms and research bases for delaying aging-related symptoms by controlling inflammation. In addition, this study still has some limitations. First, the samples were European; more studies are conducted among other population races to verify this result. Second, Sarcopenia-related syndromes and frailty were enrolled in the study for the MR analysis; research still be needed to further recognize genetic variants related to other aging-related syndromes through GWASs.

## Conclusion

In summary, we systematically assess the association between 41 circulating cytokines with the risk of aging-related syndromes from a genetic perspective. Through the MR analysis, we found some novel circulating cytokines and robust associations with aging-related syndromes and provided a research basis for anti-inflammatory pathways to reduce the risk of aging-related syndromes. Moreover, sarcopenia and frailty are defined as prototypical conditions based on a multi-morbid and functionally impaired population that require novel models of care and medical treatment.

## Data availability statement

The original contributions presented in the study are included in the article/**Supplementary Material**. Further inquiries can be directed to the corresponding author.

## Author contributions

CW: Conceptualization, Supervision, Writing – original draft, Writing – review & editing. JW: Writing – review & editing. RW: Writing – review & editing. HK: Conceptualization, Writing – review & editing. MW: Conceptualization, Data curation, Methodology, Software, Writing – review & editing.
